# Implementation of the BIOFIRE Meningitis/Encephalitis Panel: A Mixed-Methods Implementation Study in a Nonmetropolitan Tertiary Hospital

**DOI:** 10.1093/ofid/ofag240

**Published:** 2026-05-07

**Authors:** Shradha Subedi, N A Patrick Harris, Lisa Hall, L David Paterson

**Affiliations:** University of Queensland, UQ Centre for Clinical Research, Herston, Queensland, Australia; Department of Infectious Diseases, Sunshine Coast Health Service, Birtinya, Queensland, Australia; University of Queensland, UQ Centre for Clinical Research, Herston, Queensland, Australia; Pathology Queensland Central Laboratory, Royal Brisbane and Women's Hospital, Herston, Queensland, Australia; School of Public Health, University of Queensland, Herston, Queensland, Australia; ADVANCE-ID, SawSwee Hock School of Public Health, National University of Singapore, Singapore

**Keywords:** antimicrobial stewardship, diagnostic stewardship, implementation, mixed methods, rapid diagnostic tests

## Abstract

**Background:**

Meningitis and encephalitis (ME) are associated with significant morbidity and mortality. Rapid pathogen identification is essential to guide early treatment and support antimicrobial stewardship (AMS). The BIOFIRE® FILMARRAY® ME panel allows simultaneous detection of multiple pathogens from cerebrospinal fluid. This study evaluated the implementation strategy and clinician adoption of the panel in a large nonmetropolitan tertiary hospital in Australia

**Method:**

A mixed-methods study was conducted at the Sunshine Coast University Hospital following implementation of the BIOFIRE ME panel. Strategies included education sessions, testing criteria development, and continuous stakeholder engagement. Clinician knowledge, confidence, and antimicrobial decision-making practices were assessed at baseline and during implementation via surveys and semistructured interviews based on the Cabana et al conceptual framework. Quantitative data were analyzed using descriptive statistics, while qualitative data were analyzed thematically using Braun and Clarke's 5 step process.

**Results:**

A total of 213 clinicians participated in the surveys across 4 time points. Median perceived usefulness of rapid CSF PCR testing was 9/10. Confidence in ceasing antibiotics based on the assay results increased from 18% at 9 months to 52% at 12 months (*P* = .06), while confidence in ceasing antivirals remained high (85%–86%). Qualitative findings identified 5 themes influencing implementation: test-ordering practices, perceived diagnostic value, trust in results, antimicrobial stewardship, and behavioral change. Confidence in interpretation was highest where infectious diseases input was available.

**Conclusions:**

Implementation of the syndromic panel improved diagnostic efficiency and clinician confidence in antimicrobial decision making. Sustained education, multidisciplinary collaboration, and integration of stewardship frameworks were key to achieving successful adoption.

Meningitis and encephalitis (ME) are serious conditions associated with significant morbidity and mortality [1]. Rapid identification of the causative pathogen in ME is essential to enable early and targeted antimicrobial therapy [2–4], as delays increase mortality and adverse outcomes [5, 6]. Conversely, a prompt and reliable negative result enables early discontinuation of unnecessary antimicrobials, supporting antimicrobial stewardship (AMS) [7].

Nucleic acid amplification and detection tests performed on cerebrospinal fluid (CSF) in conjunction with microscopy and culture represent the standard diagnostic method for ME [8]. Many laboratories use in-house molecular tests targeting principal ME pathogens; however, developing and maintaining these assays requires advanced infrastructure, skilled personnel, and considerable resources; factors often limited outside reference laboratory settings [9].

Over the last decade, commercial molecular assays for direct CSF testing have expanded rapidly. These closed, user-friendly platforms offer rapid turnaround times (TAT) and require minimal laboratory infrastructure or technical expertise [10, 11]. Their introduction into routine laboratories has provided clinicians with timely, actionable results, significantly benefiting patient care [12], particularly in the nonmetropolitan setting.

Some commercial platforms are multiplexed, detecting up to 14 pathogens using a single test. Multiplex assays offer advantages such as reduced specimen volume requirements, important in pediatrics, and the ability to streamline test selection through a syndromic framework. Traditional workflows rely on multiple singleplex PCR assays performed sequentially, contributing to delays and increased costs. Syndromic multiplex panels targeting key central nervous system (CNS) pathogens can simplify diagnostic algorithms and substantially shorten CSF testing TATs [13–15].

Despite these benefits, syndromic panels also pose challenges, including high cost and the need for careful implementation to ensure optimal clinical outcomes [16, 17]. In the setting of syndromic panels, implementation refers to the integration of multiplex panel into both clinical and laboratory workflows to facilitate timely and accurate identification of pathogens and resistance determinants. This involves several elements, including selection of an assay aligned with local epidemiology and clinical priorities, engagement across multidisciplinary teams, compliance with regulatory standards, integration within laboratory processes, and consideration of financial and resource implications [18].

Measuring quantitative outcomes such as TAT, duration of antimicrobials, or cost-effectiveness analysis is important, but often fails to capture the behavioral and contextual factors that determine whether a diagnostic assay achieves sustainable clinical impact. An implementation evaluation exploring clinician attitudes, decision-making processes, and workflow barriers provides a complementary approach to understanding these dimensions. Such insights are critical to refining implementation strategies, promoting adoption, and informing broader translational frameworks for diagnostic stewardship [19–21].

In this study, we describe strategies used to implement the BIOFIRE® FILMARRAY® ME Panel in a large nonmetropolitan Australian hospital. The implementation strategy was guided by principles of diagnostic stewardship and behavior change frameworks addressing clinician knowledge, attitudes, and practice barriers. We outline the laboratory and clinical implementation processes and present clinician feedback gathered through serial surveys and interviews, providing a mixed-methods evaluation of the implementation process and its impact on diagnostic practice. This approach aligns with standards for reporting implementation studies (StaRI) guidance by explicitly addressing the intervention, the implementation strategy, and the contextual factors influencing outcomes.

## METHODS

### Context and Location

The study was conducted at the Sunshine Coast University Hospital (SCUH) located in the Sunshine Coast region of Queensland, Australia. Sunshine Coast University Hospital is a 738-bed tertiary teaching hospital that provides specialist services including medical, cancer, surgical, maternity, pediatric, and mental health services to the Sunshine Coast and Gympie regions.

**Figure 1.**
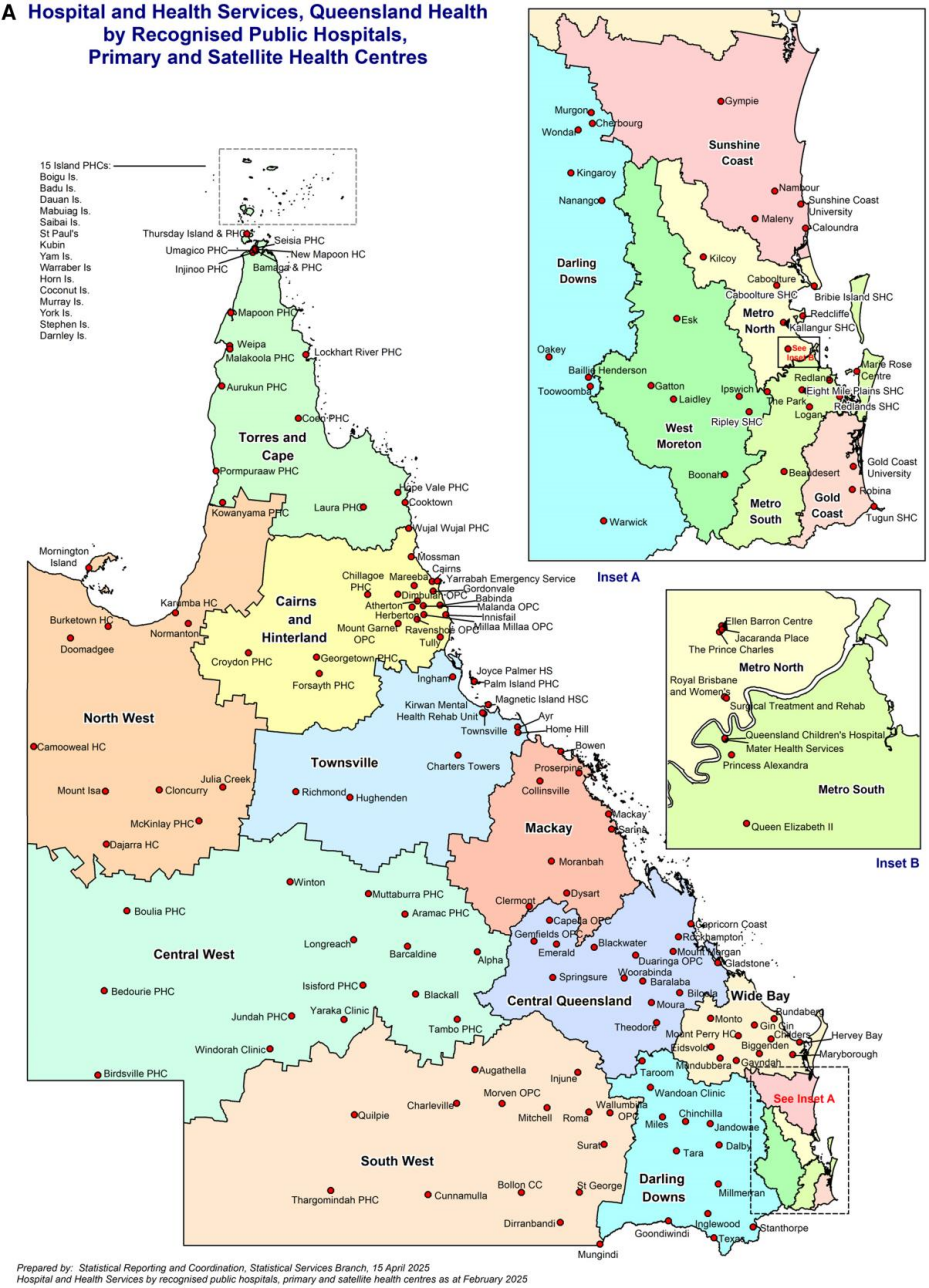

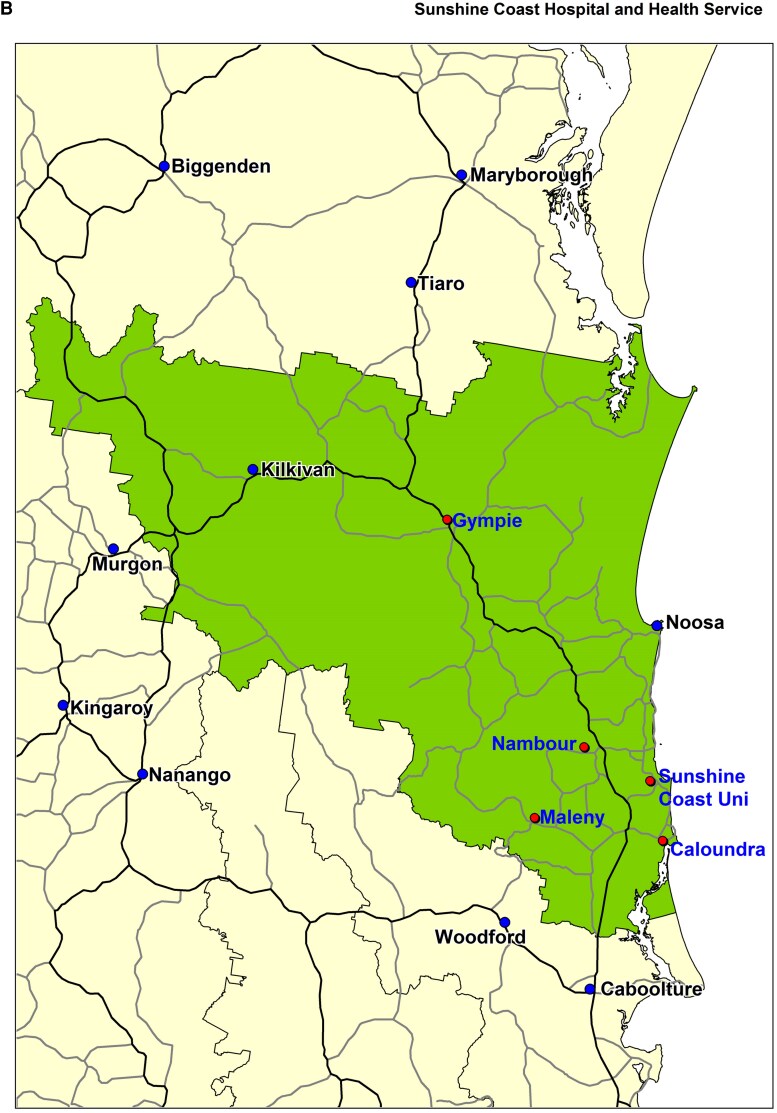
(*A* and *B*) Geographic context of the study and laboratory network.

The Pathology Queensland microbiology laboratory located at SCUH provides microbiology diagnostic services to patients admitted to SCUH and other public hospitals in the region, including Nambour, Maleny, Caloundra, and Gympie Hospitals (Figures 1*A* and 1*B*) [22]. The hospitals within the region (∼1000 beds in total) serve a population of ∼460 000. While the SCUH microbiology laboratory provides diagnostic services to other smaller hospitals in the region, the qualitative and survey components of this study were restricted to clinicians based at SCUH. Prior to 2023, CSF molecular testing requests were referred to the reference Pathology Queensland Central Laboratory located in Brisbane, Queensland (∼1.5 h drive away) with a TAT of ∼24 h from CSF collection. For select requests eg, *Listeria monocytogenes*, *E. coli*, or Group B Streptococcus PCR, the samples were sent away to an additional external laboratory with a TAT of upto 7 days.

The BIOFIRE ME panel (BIOFIRE Diagnostics, LLC, Salt Lake City, UT) was introduced at SCUH in February 2023. Following an initial transition period, during which the assay was used selectively for neonates and patients with suspected ME due to *L*. *monocytogenes,* the assay was then fully implemented in September 2023 for all patients with suspected ME.

### Implementation Strategies

The implementation strategies employed in this study were divided into 2 main components: laboratory and clinical implementation, which are described below. These strategies broadly included education and training, development of a testing framework, and continuous stakeholder engagement throughout the study period.

### Laboratory Implementation

Laboratory implementation of the BIOFIRE ME panel began with a literature review to identify strengths, limitations, and known sensitivity or specificity issues, particularly for HSV-1, *Cryptococcus* and *H. influenzae* targets [23, 24]. Mitigation strategies were incorporated into testing algorithms, and all results were reviewed by a clinical microbiologist to ensure concordance with patient presentation. These strategies are summarized in Table 1.

**Table 1.** Bio Fire ME Panel: Laboratory Implementation Strategies

Domain	Characteristics	Mitigating Strategies, if Required
Regulatory approval	ARTG in place Laboratory verification	Laboratory verification to meet NPAAC standards in SCUH microbiology laboratory
Test performance	Low sensitivity reported for some targets, eg, HSV-1, Cryptococcus	Clinician education All negative results reviewed by clinical microbiologist Strategies in place for suspected false negatives: eg, request Cryptococcus antigen testing separately
Test performance	Low specificity for some targets, eg *Streptococcus pneumoniae*	All positive results reviewed by clinical microbiologist and discussed with treating clinicians. Measures to reduce contamination in line with other laboratory tests
Cost	High cost of the assay and consumables	Diagnostic stewardship Thorough health economic evaluation
Diagnostic stewardship	Possible overuse of the assay	Laboratory testing criteria^a^, test monitoring, clinician education and feedback

Abbreviations: ARTG, Australian register of therapeutic goods; HSV-1, Herpes simplex virus 1; NPAAC, National Pathology Accreditation Advisory Council; PCR, polymerase chain reaction; SCUH, Sunshine Coast University Hospital.

^a^Routine CSF PCR testing required abnormal cell count/biochemistry; however, testing was performed on all requests for ICU patients and children <12 m irrespective of CSF biochemistry or microscopy.

The assay was registered in the Australian Register for Therapeutic Goods (ARTG), fully verified to meet accreditation standards, and introduced with defined testing hours, staff training, and measures to reduce contamination. The assay was made available 7 days per week between 8:00 am and 4.30 pm, providing optimal testing capacity to support timely decision making. Laboratory testing criteria were aligned with the existing state-based guideline for molecular testing of CSF. The high cost of the consumables was recognized as a key limitation of the assay, and a testing strategy was put in place after discussion with all the relevant stakeholders and a plan was developed to conduct a comprehensive health economic evaluation (Table 2).

**Table 2.** Summary of Quantitative Survey Results

	Survey 2	Survey 3; N (%)	Survey 4	*P* Value
Total number of respondents	40	44	30	0.73
Trust the results all the time	…	10 (32)	12 (50)	
Trust the results most of the time	…	19 (61)	12 (50)	
Trust the results some of the time	…	1 (2)	0	
Above questions not applicable	…	14 (32)	6 (20)	
Comfortable ceasing antibiotics all the time	…	6 (18)	12 (52)	0.06
Comfortable ceasing antibiotics most of the time	…	20 (61)	9 (39)	
Comfortable ceasing antibiotics some of the time	…	7 (21)	2 (9)	
Above questions regarding antibiotics not applicable	…	11 (25)	7 (23)	
Comfortable ceasing antivirals all the time	…	12 (38)	10 (43)	0.36
Comfortable ceasing antivirals most of the time	…	15 (47)	10 (43)	
Comfortable ceasing antivirals some of the time	…	5 (16)	3 (13)	
Above questions regarding antivirals not applicable	…	12 (27)	7 (23)	
Know who to contact to discuss results	27 (77)	33 (79)	24 (86)	0.46
Do not know who to contact to discuss results	8 (23)	9 (21)	4 (14)	
Above questions not applicable	5 (13)	2 (5)	2 (7)	
Implementation working well	24 (60)	…	24 (80)	0.07
Unsure or question not applicable	16 (40)	…	10 (20)	

### Clinical Implementation

Before implementation, the principal investigator (S.S.) met with clinicians from pediatrics, infectious diseases (ID), neurology, emergency medicine, intensive care, and general medicine to introduce the assay and its clinical role. Over 6 months, S.S. delivered presentations explaining study aims, methodology, assay performance, and limitations. A summary handout (Supplementary material 1) was distributed and recirculated regularly. These sessions encouraged multidisciplinary engagement, clarified expectations, and allowed clinicians to raise questions or concerns that informed the implementation process. Key discussion themes arising from these meetings are summarized in the results section.

### Implementation Evaluation

Progress was monitored through quarterly assessments of key implementation milestones and evaluations of clinicians’ understanding of the assay's value and result interpretation through serial surveys. An Organizational Readiness Assessment was developed by L.H. for use at the beginning of the implementation phase and updated by S.S. on a quarterly basis. Identified gaps were addressed on an iterative basis to ensure sustained implementation progress and alignment with project objectives (Supplementary material 2).

Clinician surveys were administered during implementation in paper format to the same key clinical groups (general medicine, ID, neurology, pediatrics, emergency medicine, and intensive care). Survey instruments were developed by S.S. and reviewed by L.H. based on the core constructs in the Cabana et al conceptual framework of provider barriers to following clinical practice guidelines [25]. The final survey instruments were reviewed and approved by the rest of the study team. Surveys were conducted anonymously and respondents were not tracked across time points. Therefore, the analysis treated each survey wave as a repeated cross-sectional sample. Semistructured face-to-face interviews were conducted by S.S. with 5 consultants (general medicine, ID, ICU, pediatrics, and neurology) at the end of the implementation phase (Supplementary material 3). Interview questions were prepared by S.S. and reviewed by L.H. and were based on the Cabana framework and survey findings. The interview lasted between 8 and 21 min (median duration 10 min). These interviews explored clinicians’ perspectives on the successes and challenges of the implementation process and gathered feedback to inform future testing pathways and optimization strategies (Supplementary material 4).

### Data Analysis

Survey results were analyzed using descriptive statistics. Categorical variables were reported as frequencies and percentages, and continuous variables as means or medians. The χ^2^ testing assessed statistical significance between categorical variables, with *P* < .05 considered significant. Statistical analyses were performed using Microsoft Excel and Stata V18. Qualitative data from meetings and interviews were analyzed thematically using and Clarke's 6-step framework [26], which included data familiarization (reviewing transcripts, minutes, and survey responses), generating initial codes, organizing codes into themes, reviewing and defining themes, and producing a summary report with illustrative examples. Initial coding was performed manually by the primary investigator (S.S.). The coding framework and emerging themes were subsequently reviewed against transcripts with a second investigator (L.H.) to enhance analytical rigor and minimize potential investigator bias. Given the relatively small dataset, qualitative analysis software was not used. The study was reported in accordance with the StaRI guidelines to ensure transparent reporting of both the diagnostic intervention and the implementation strategies [27] (Supplementary 5).

### Ethics

Ethics approval for this study was obtained from the Metro North Hospital and Health Service Human Research and Ethics Committee (HREC/2022/QPCH/78939).

## RESULTS

### Quantitative Results

**Figure 2.**
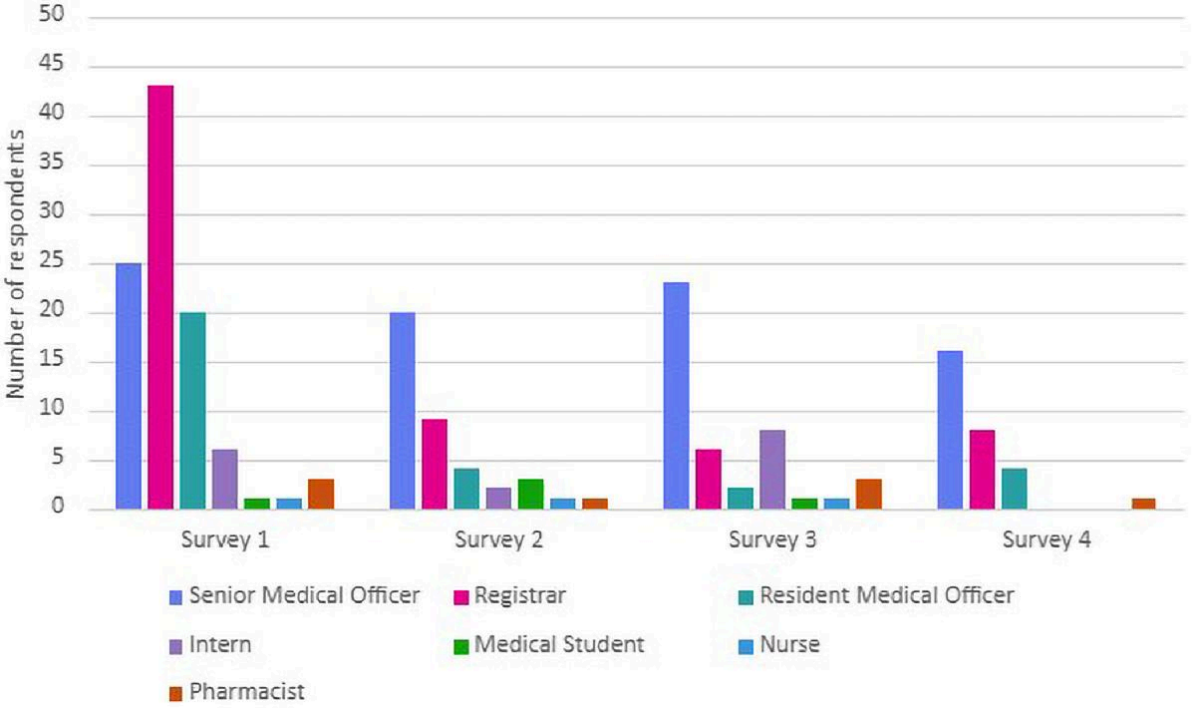
Distribution of survey respondents by role.

A total of 99 clinicians responded to Survey 1, 40 completed survey 2 (5 months postimplementation), 44 completed survey 3 (9 months postimplementation), and 30 completed survey 4 (12 months postimplementation). Respondent distribution across the 4 surveys is shown in Figure 2. In response to the preimplementation question assessing the perceived usefulness of rapid diagnostic PCR testing on CSF (rated on a 10-point Likert scale, with 1 being least useful and 10 being most useful), the median score was 9 [IQR 8–10], reflecting a high level of perceived clinical utility. Most clinicians (77%) reported ordering PCR testing upfront before reviewing CSF microscopy or biochemistry, while 16% preferred to wait for those results.

Following implementation, clinician confidence and antimicrobial decision-making improved over time. At 5 months (Survey 2), 24 out of the 40 reported that the implementation was working well, increasing to 24 out of 30 at 12 months (*P* = .07). Trust in the assay results increased from 32% (trusting results “all the time”) at 5 months to 50% at 12 months (*P* = .73).

Comfort with ceasing antimicrobials also improved (Table 2). At 9 months, 78% were comfortable ceasing antibiotics “all or most of the time” based on the results provided by the assay, which increased to 91% by 12 months (*P* = .06), suggesting a trend toward greater clinician comfort. For antivirals, there was a high confidence in ceasing antivirals all or most of the time, at 85% at 9 months and 86% at 12 months (*P* = .36). Knowledge of who to contact for result interpretation remained consistently high (≥75%) across all time points (*P* = .46)

### Qualitative Results

Qualitative analysis of clinician interviews, descriptive answers on the survey report and meeting discussions identified 5 overarching themes that reflected before and after implementation of onsite syndromic panel implementation: (1) test-ordering criteria, (2) perceived benefits of rapid diagnostic tests, (3) trust in the results provided by the assay and actioning on the results (4) impact on AMS, and (5) perceptions of assay sensitivity and specificity.

### Test-Ordering Criteria

Clinicians reported variable test-ordering practices prior to implementation, with most preferring upfront PCR rather than waiting for microscopy or biochemistry. Decisions were influenced by age, presentation, and disease severity. Pediatric and neonatal clinicians noted reduced microscopy sensitivity after antibiotics reflected in comments such as, “*If there were high suspicion of meningitis, I would request PCR upfront prior to microscopy and biochemistry”* and “*Cell counts can be benign even in bacterial meningitis.”* Some clinicians preferred retrospective PCR depending on initial results and clinical progress, whereas others supported laboratory-defined criteria with discussion if a request did not meet indications. An ED consultant suggested the laboratory “*cancel if PCR is not indicated, after discussing with the requesting clinician.”* ICU clinicians preferred testing whenever a lumbar puncture was performed in a patient already receiving antimicrobials: “*If we have started someone on antimicrobials and performed lumbar puncture, we would like to see a PCR result.”* ID clinicians incorporated microscopy findings and clinical risk factors into their decisions.

### Perceived Benefits of Rapid Diagnostic Test

Clinicians identified substantial benefits associated with onsite multiplex testing, particularly regarding its potential impact on timeliness of diagnosis, treatment optimization, and length of stay. Several participants emphasized that rapid identification of viral meningitis could prevent unnecessary antimicrobial therapy. A pediatric registrar highlighted, “*Many of our patients have viral meningitis with normal cell counts, and having a rapid diagnosis may change length of stay and treatment given.”* Postimplementation feedback reinforced these perceptions, with ICU and neurology consultants noting the test's value in rapidly ruling out infectious causes and supporting earlier management of alternative diagnoses such as autoimmune encephalitis. One clinician noted, “*The rapid turnaround means patients potentially get at least three fewer doses of acyclovir at the very least.”* Another commented, “*Earlier immunotherapy for autoimmune encephalitis leads to better long-term outcomes.*” The inclusion of additional targets not available on in-house assays, such as *L*. *monocytogenes*, *E. coli*, and Group B Streptococcus was also regarded as beneficial.

### Clinician Trust in the Assay and Actioning of Results

Clinicians generally expressed high levels of trust in the assay, particularly when results were supported by established ID consultation pathways. One ICU consultant stated, “*I’m reasonably confident because we have additional backup and discussion with the ID team.”* Nonetheless, clinical context remained central to interpretation. In complex or high-risk cases, clinicians often sought to correlate results with clinical presentation and imaging findings, and in some instances, repeat testing was considered, particularly if HSV-1 encephalitis was suspected. On reflection, 1 participant described a subtle cognitive bias toward single-target assays, perceiving them as more specific than multiplex panels despite comparable performance, “*When you do a PCR for a single organism, it feels more precise than a multiplex panel, even though the sensitivity and specificity might be similar.*”

### Impact on AMS

Participants consistently linked the introduction of onsite syndromic testing with improvements in AMS, particularly through reduced duration of empiric therapy in low-risk patients. As one ICU clinician noted, “*It absolutely helps, especially for the low-risk patients where you can stop antivirals and focus on other diagnoses*.” However, some clinicians observed that behavioral and educational barriers limited the full realization of AMS benefits. A neurology consultant reported, “*My juniors have been a bit hit and miss, some continued acyclovir even after the BioFire came back negative*.” Another ID consultant suggested that confusion about assay workflows may have contributed, noting, “*Maybe people thought they were still going to get an in-house result because it looks different*.” Participants also highlighted the importance of structured handovers and explicit stop plans to ensure antimicrobial discontinuation after negative results, while acknowledging that practice change takes time, as reflected by 1 neurology consultant who noted, “*Human behaviour takes some time to change*.”

### Perceptions of Assay Sensitivity and Specificity

Clinicians generally viewed the assay as highly reliable, with rare discordant results noted. An ID consultant recalled, “*A pneumococcal result was negative on BioFire but positive on the specific assay… these are rare, and all tests have sensitivity and specificity issues.”* Some participants noted that understanding of assay performance varied among clinical teams, particularly among nonspecialists, with 1 consultant suggesting that “*the assay is probably a bit more complex to understand for a non-ID, non-microbiology clinician.”*

## DISCUSSION

Implementation of an onsite multiplex syndromic panel for CSF testing was associated with strong clinician engagement and progressively increasing confidence in the assay and its clinical impact. Across the 12-month period, clinicians consistently expressed trust in results and comfort modifying antimicrobial therapy based on panel findings, reflecting gradual behavioral adaptation. The preimplementation survey showed high baseline support, with a median usefulness score of 9/10.

Before implementation, clinicians described variability in CSF test-ordering practices. Most clinicians (77%) preferred to request PCR testing upfront, before reviewing CSF microscopy and biochemistry results. This may reflect a clinical workflow practice, or concern regarding the reliability of microscopy and biochemistry results in confidently excluding ME. Following implementation, both quantitative and qualitative findings demonstrated that the syndromic panel was perceived to improve diagnostic efficiency, enable earlier de-escalation of empiric therapy, and facilitate earlier consideration of alternative diagnoses, particularly autoimmune encephalitis. These findings align with prior evaluations of the BIOFIRE ME Panel, demonstrating reduced TATs, earlier pathogen identification, and shorter durations of antimicrobial therapy [17, 28–30].

Clinician confidence in ceasing antivirals remained consistently high at both 9- and 12-months (85% and 86%, respectively), consistent with results showing reduced acyclovir following panel introduction [28, 30]. In contrast, confidence in stopping antibiotics increased over time, with 52% comfortable stopping antibiotics “all the time” at 12 months, compared with 18% at 9 months (*P* = .06). This progressive improvement likely reflects growing clinician familiarity and trust in the assay and highlights the fact that meaningful practice change evolves gradually. Similar temporal patterns have been observed following implementation of respiratory syndromic panels. One study demonstrated progressive reductions in antibiotic duration across successive implementation periods, suggesting increasing clinician confidence in test results over time [31]. In another study, time series analysis showed that the introduction of syndromic panels (respiratory and ME) alongside clinical guidelines reduced ancillary testing and antibiotic use, with continued declines over time consistent with ongoing clinician learning and adaptation [32]. These observations support the concept that the full benefits of rapid diagnostics may only emerge after an early adoption phase, an important consideration when designing implementation studies and evaluating outcomes. Prior to implementation, clinicians described variable ordering practices influenced by uncertainty about microscopy sensitivity, illness severity, and workflow considerations. One clinician noted that “*the lab cancel if PCR is not indicated, after discussing with the requesting clinician*,” highlighting the need for risk-based laboratory testing criteria.

Trust in the assay was context-dependent, particularly in high-risk cases or when HSV-1 encephalitis was suspected. To mitigate concerns about false negatives, laboratories should have clear pathways for managing patients with high clinical suspicion of ME and a negative BIOFIRE result. Some clinicians described a cognitive bias toward single-target PCRs, perceiving them as more specific than multiplex panels despite comparable performance characteristics. These findings mirror previous reports that highlight the influence of cognitive heuristics and risk aversion in diagnostic decision making [33]. Addressing such biases will require structured education and clear communication of assay performance.

The results also reinforce the importance of multidisciplinary collaboration. Confidence in assay interpretation was greatest when ID input was available, such as in ICU. This is consistent with evidence that structured ID involvement enhances diagnostic stewardship and clinician adherence to test results. While respondents consistently associated the assay with improved AMS, the qualitative data revealed barriers to sustained behavior change. Some clinicians continued empiric antivirals or antibiotics despite negative results. Addressing these barriers may require integration of automated stewardship prompts, decision-support tools, and reinforcement of “stop” protocols during clinical handovers [14, 34].

In terms of test utilization, the volume of BIOFIRE ME Panel testing increased in the pediatric population, reflecting growing clinical uptake (12-month usage: adult 117 preimplementation and 114 postimplementation; pediatrics 74 preimplementation and 106 postimplementation, unpublished data). Testing criteria generally required CSF pleocytosis, and when criteria were not met, requests were reviewed by a clinical microbiologist, with decisions for further testing or rejection made based on clinical judgment; formal data on rejected tests were not collected. The appropriateness of all requests was therefore not systematically assessed. A health economic evaluation of the BIOFIRE ME Panel is also underway, with results still being analyzed, which will provide further insight into the cost-effectiveness and resource implications of implementation.

This mixed-methods study provides detailed insights into clinician attitudes and behaviors surrounding implementation of an onsite CNS syndromic panel. The combination of sequential surveys, interviews, and meeting data offers a comprehensive view of implementation dynamics. However, the study has several limitations: Participation in the surveys was voluntary and anonymous, which may have introduced response bias, as clinicians who chose to respond could have been more engaged with or supportive of the BIOFIRE ME panel than nonrespondents. Because the surveys were anonymous, we were unable to track repeated participation across waves or determine the total number of nonrespondents, preventing sensitivity analyses to explore potential nonresponse bias. Surveys relied on self-reported rather than objective prescribing data, and declining participation in later waves suggested some survey fatigue. Additionally, this evaluation was conducted pragmatically in a single nonmetropolitan tertiary hospital without piloting or iterative refinement of the survey instrument. As such, findings are context-specific and hypothesis-generating, providing insights that may inform future, more rigorous implementation studies in other settings. Despite these limitations, the mixed-methods approach allowed comprehensive exploration of clinician attitudes, behaviors, and contextual factors influencing adoption of the BIOFIRE ME panel.

## CONCLUSION

The implementation of the BIOFIRE Meningitis/Encephalitis Panel into clinical practice demonstrated that rapid syndromic molecular testing could enhance diagnostic turnaround, clinician confidence, and antimicrobial optimization in the management of ME. Successful adoption required robust governance, clinician engagement, and integration with AMS frameworks. Ongoing education, audit, and feedback mechanisms remain essential to maintain appropriate test utilization and maximize the clinical impact of syndromic diagnostic panels.

## Supplementary Material

ofag240_Supplementary_Data

## Supplementary Data

Supplementary materials are available at *Open Forum Infectious Diseases* online. Consisting of data provided by the authors to benefit the reader, the posted materials are not copyedited and are the sole responsibility of the authors, so questions or comments should be addressed to the corresponding author.
