# Supplementary material for: Implementation of the BIOFIRE Meningitis/Encephalitis Panel: A Mixed-Methods Implementation Study in a Nonmetropolitan Tertiary Hospital
Source: Open Forum Infect Dis. 2026 May 7;13(5):ofag240. doi: 10.1093/ofid/ofag240 (PMC13152011; doi:10.1093/ofid/ofag240)
Supplement: ofag240_Supplementary_Data [file ofag240_supplementary_data.zip › Supplementary material 1.pdf]

BioFire ME Study: Use of an onsite Rapid PCR assay (BioFire ME panel) in patients with suspected meningitis/ encephalitis-Clinical and Health Economic analysis

Summary

This study will evaluate the clinical and health economic benefit of having an onsite rapid diagnostic panel (BioFire ME panel) for pathogen detection from Cerebrospinal Fluid (CSF) in patients with suspected meningitis/encephalitis.

What is the BioFire ME panel?

BioFire ME panel is a rapid, commercial multiplex syndromic panel for meningitis/encephalitis that targets 14 most common bacteria, viruses and yeasts that cause community acquired meningitis and encephalitis.

Pathogens targeted by the assay are:

**Bacteria**
Escherichia coli K1
Haemophilus influenzae
Listeria monocytogenes
Neisseria meningitidis
Streptococcus agalactiae
Streptococcus pneumoniae
**Viruses**
Cytomegalovirus (CMV)
Enterovirus (EV)
Herpes simplex virus 1 (HSV1)
Herpes simplex virus 2 (HSV2)
Varicella zoster virus (VZV)
HHV-6
**Yeast**
Cryptococcus (C.neoformans/ C.gattii)

What are the limitations of the assay?

The assay does not cover some pathogens that can occasionally cause meningitis/ encephalitis. These include, but are not limited to Mycobacterium tuberculosis, Japanese encephalitis and other arthropod-borne viruses, rickettsia, syphilis etc. The assay only targets pathogens that cause community acquired meningitis/encephalitis. If your patient has suspected healthcare acquired meningitis, please discus with the Infectious Diseases physician or registrar on call.

Published data demonstrate that the assay has a lower sensitivity for the detection of Cryptococcus DNA, compared to Cryptococcal antigen lateral flow assay. For patients with high clinical suspicion of Cryptococcus meningitis, please request Cryptococcal antigen separately. Detection of HHV-6 and CMV may signify latency rather than true infection and clinical correlation is recommended.

In rare instances, the assay has been reported to miss HSV1, particularly if the CSF is collected early in the course of illness. If your patient has a high clinical suspicion of HSV encephalitis and the HSV PCR is negative, please discuss with the ID physician on call or the Clinical Microbiologist on **(07) 5202 8831.**

For questions regarding this study, please contact the Principal Investigator:

Dr Shradha Subedi
Infectious Diseases Physician
and Clinical Microbiologist

Sunshine Coast Hospital and Health Service
E: shradha.subedi@health.qld.gov.au
