# Supplementary material for: Implementation of the BIOFIRE Meningitis/Encephalitis Panel: A Mixed-Methods Implementation Study in a Nonmetropolitan Tertiary Hospital
Source: Open Forum Infect Dis. 2026 May 7;13(5):ofag240. doi: 10.1093/ofid/ofag240 (PMC13152011; doi:10.1093/ofid/ofag240)
Supplement: ofag240_Supplementary_Data [file ofag240_supplementary_data.zip › Supplementary material 2.docx]

## Supplementary material 2: Organisational Readiness Assessment; ME clinical study

| **ON TRACK** | **IMPROVEMENT REQUIRED** | **AT RISK** | **COMPLETE** | **NOT APPLICABLE** |
| --- | --- | --- | --- | --- |
| Organisation currently on track to achieving a state of readiness to support the project effectively. | Potential for full program benefits not to be achieved without intervention, however opportunities exist to strengthen this area. | Current organisational state is likely to impact the successful outcome of the project. Significant work required in this area prior to  undertaking project activities. | Evidence can be provided to support the organisational readiness in this area and for the project to operate effectively. | Outlined requirement is not applicable for this quarter. |

| **Indicator** | **Criteria** | **2023/2024** | | | |
| --- | --- | --- | --- | --- | --- |
|  |  | **Q1** | **Q2** | **Q3** | **Q4** |
| **Organisation** | | | | | |
| Organisation support | There is documented executive level support for the project. |  |  |  |  |
| Governance | Program activities and outcomes will be reported to all relevant stakeholders. |  |  |  |  |
| Department support | There is support from relevant departments, e.g., the Emergency Department, Intensive Care Unit, general medicine, neurology,  paediatrics, and neonatology |  |  |  |  |
| **People** | | | | | |
| Leadership | A steering group, including staff from relevant departments at  various levels, has been formed and are committed to remaining  engaged throughout the project. |  |  |  |  |
| Stakeholders | Stakeholders from relevant departments at various levels are aware of and support the program. |  |  |  |  |

| Project leaders and champions | A project research coordinator (e.g. microbiologist, nurse) and site principal investigator have been identified to support  implementation and data collection at the site. |  |  |  |  |
| --- | --- | --- | --- | --- | --- |
|  | There is a plan for regular communication between the steering committee and key stakeholders |  |  |  |  |
| Consumers | Consumer Research Engagement Group (CREG) − Consumer  representatives have been identified and are engaged with the program. |  |  |  |  |
| **Policy** | | | | | |
| Policies and procedures | Testing and diagnostic referral guidelines are available at the site and any areas requiring local adaptation have been identified. |  |  |  |  |
|  | Existing policies regarding treatment of infections have undergone review with any changes required identified. |  |  |  |  |
|  | Changes in local policies required are made available to the steering group. |  |  |  |  |
|  |  |  |  |  |  |
|  |  |  |  |  |  |
| Existing training infrastructure | Policies to support clinician education at the site are available and have been identified |  |  |  |  |
|  | Project champions have received education on the BioFire ME panel and changes to diagnostic testing guidelines |  |  |  |  |
|  |  |  |  |  |  |

| **Infrastructure** | | | | | |
| --- | --- | --- | --- | --- | --- |
| Electronic medical records | Electronic medical records are available to relevant staff. |  |  |  |  |
|  | Hard copy policy and program details are readily available if  electronic systems are not. |  |  |  |  |
| Patient review | Adequate infrastructure for data collection is in place (including  medical record, computer and internet access). |  |  |  |  |
| Monitoring | Capacity for the collection of data relating to key performance indicators for program efficacy and safety. |  |  |  |  |
|  | Electronic medical records systems are sufficient for collection of  project data and performance indicators. |  |  |  |  |
| **Evaluation** | | | | | |
| Safety | Critical performance indicators relating to patient safety will be captured (missed infections, adverse events). |  |  |  |  |
|  | Formal analysis will be completed for all patients affected by adverse events in accordance with local policies, and relevant information provided to the steering group as permitted. |  |  |  |  |
| Efficiency | Other performance indicators will also be monitored   - TAT to pathogen detection - All clinical failure (death or adverse outcome) - In−hospital all−cause mortality - Duration of antimicrobial therapy - Costs per patient |  |  |  |  |
| Quality of life | Capacity exists to obtain consent from patients and their carers/guardians for quality of life and health economics in a timely manner. |  |  |  |  |
| Economic outcomes | Required data can be collected to enable local health information systems to provide cost data. |  |  |  |  |

| Feedback to consumers and staff | Processes for regular staff and consumer feedback have been identified, including capacity for reporting of performance  indicators. |  |  |  |  |
| --- | --- | --- | --- | --- | --- |
| Feedback from consumers and staff | Opportunities exist and are utilised to process feedback from stakeholders regarding performance indicators. |  |  |  |  |
|  | Policy and education materials can be updated in response to feedback. |  |  |  |  |
| Ethics | Ethics and governance applications submitted to facilitate data collection. |  |  |  |  |
