# Supplementary material for: Implementation of the BIOFIRE Meningitis/Encephalitis Panel: A Mixed-Methods Implementation Study in a Nonmetropolitan Tertiary Hospital
Source: Open Forum Infect Dis. 2026 May 7;13(5):ofag240. doi: 10.1093/ofid/ofag240 (PMC13152011; doi:10.1093/ofid/ofag240)
Supplement: ofag240_Supplementary_Data [file ofag240_supplementary_data.zip › Supplementary material 3.docx]

Survey 1

1. I am

SMO

Registrar

RMO

Intern

Medical Student

Nurse

Pharmacist

1. The department/hospital I currently work is:    ________________

1. I think the availability of onsite rapid diagnostics (PCR) on CSF for patients with suspected meningitis/encephalitis will be helpful for our HHS (1=unhelpful, 10= very helpful)

1    2    3    4    5    6    7    8    9    10

1. I would like to order PCR on the CSF upfront, prior to the results of the microscopy/biochem are available

Yes

No

N/A

Date survey filled    ________________

Survey 2

1. I am

SMO

Registrar

RMO

Intern

Medical Student

Nurse

Pharmacist

1. The department/hospital I currently work is:    ________________

1. I believe the implementation of the BIOFIRE CSF panel in this HHS is working well

True

False

Don’t know

N/A

Comments: ________________________________

1. I trust the results provided by the BioFire panel

I trust the results all the time

I trust the results most of the time

I trust the results sometime

I never trust the results

N/A

Comments: ____________________________________

1. I know who to contact if I have any questions regarding the interpretation of results or further management of patients with suspected meningitis and encephalitis

True

False

Don’t know

N/A

Comments: ___________________________________

Date survey filled    ________________

Survey 3

1. I am

SMO

Registrar

RMO

Intern

Medical Student

Nurse

Pharmacist

1. The department/hospital I currently work is:    ________________

1. Have you used the BioFire ME CSF panel?

Yes

No

Not sure

Not applicable

1. I feel comfortable ceasing antibiotics based on the results of the BioFire CSF panel

Yes, all the time

Yes, most of the time

Yes, some of the time

No, never

Not applicable

Comment   ___________________________________

1. I feel comfortable ceasing antivirals based on the results of the BioFire CSF panel

Yes, all the time

Yes, most of the time

Yes, some of the time

No, never

Not applicable

Comment    _________________________________

1. Do you trust the results provided by the BioFire panel

I trust the results all the time

I trust the results most of the time

I trust the results sometime

I never trust the results

Not applicable

Comments: ____________________________________

1. I know who to contact if I have any questions regarding the interpretation of results or further management of patients with suspected meningitis and encephalitis

True

False

Don’t know

Not applicable

Comments: ___________________________________

Survey 4

1. I am

SMO

Registrar

RMO

Intern

Medical Student

Nurse

Pharmacist

1. The department and hospital I currently work at is:    ________________

1. I have used the BioFire ME panel before

True

False

Not applicable

1. I believe the implementation of the BIOFIRE CSF panel in this HHS is going well

True

False

N/A

Comments: ________________________________

1. I trust the results provided by the BioFire panel

All the time

Most of the time

Some of the time

Never

Not applicable

Comments: ____________________________________

1. I feel comfortable ceasing antibacterial agents on my patients based on the results provided by the BioFire ME panel.

All the time

Most of the time

Some of the time

Never

Not applicable

1. I feel comfortable ceasing antiviral agents on my patients based on the results provided by the BioFire ME panel

All the time

Most of the time

Some of the time

Never

Not applicable

1. I know who to contact if I have any questions regarding the interpretation of results or further management of patients with suspected meningitis and encephalitis

True

False

Not applicable

Comments: ___________________________________

Date survey filled    ________________
