# Supplementary material for: Implementation of the BIOFIRE Meningitis/Encephalitis Panel: A Mixed-Methods Implementation Study in a Nonmetropolitan Tertiary Hospital
Source: Open Forum Infect Dis. 2026 May 7;13(5):ofag240. doi: 10.1093/ofid/ofag240 (PMC13152011; doi:10.1093/ofid/ofag240)
Supplement: ofag240_Supplementary_Data [file ofag240_supplementary_data.zip › Supplementary material 4.docx]

**Semi structured interview Topic Guide**

Thank you for participating in our study. Do you have any questions from the information sheet?

Background

Our aim is to understand the clinicians’ perspective on implementation of a rapid diagnostic test (BioFire ME panel) for the diagnosis of meningitis/encephalitis at the Sunshine Coast Hospital and Health Service.

**Consent form**

**Interview**

Recording now….

**Questions- demographics**

1. What is your job description?
2. Gender- male or female?
3. How many years of experience do you have in your current role?
4. Do you treat or cater for- adult/paediatric/both patients?

**Questions- implementation**

1. What are your thoughts on having an onsite rapid diagnostic PCR panel (BioFire ME) for detection of pathogens from CSF long term. What are some things you liked about this assay? What are some of the things you didn’t like about the assay?
2. Can you recall any examples in the past 12 months of where a rapid PCR result (positive or negative) may have led to improved patient outcomes?
3. Were there any instances where there were potential adverse patient outcomes from using this test?
4. How confident do you feel about the results provided by the assay? Are there any particular targets that you are concerned about? Who would you ring to get further advice?
5. Do you feel comfortable ceasing antibiotics based on results of the assay? Please explain
6. Do you feel comfortable ceasing antiviral based on results of the assay? Please explain
7. Our preliminary results show that whilst the acyclovir use was lower in the BioFire group, the ceftriaxone use was higher, do you have any explanations for why that may be?
8. With regards to your patient group, which patients would you request BioFire testing:

Abnormal white cell count

Abnormal white cell count or biochemistry

Any suspected cases irrespective of cell count or chemistry results

Age dependent cut off

Other
